# Instrument specific use-dependent plasticity shapes the anatomical properties of the corpus callosum: a comparison between musicians and non-musicians

**DOI:** 10.3389/fnbeh.2014.00245

**Published:** 2014-07-16

**Authors:** Henning Vollmann, Patrick Ragert, Virginia Conde, Arno Villringer, Joseph Classen, Otto W. Witte, Christopher J. Steele

**Affiliations:** ^1^Department of Neurology and Clinic for Cognitive Neurology, Max Planck Institute for Human Cognitive and Brain Sciences, University Hospital LeipzigLeipzig, Germany; ^2^Department of Neurology, University of LeipzigLeipzig, Germany; ^3^Danish Research Centre for Magnetic Resonance, Centre for Functional and Diagnostic Imaging and Research, Copenhagen University Hospital HvidovreCopenhagen, Denmark; ^4^Berlin School of Mind and Brain, Humboldt UniversityBerlin, Germany; ^5^Department of Neurology, Friedrich Schiller University JenaJena, Germany

**Keywords:** use-dependent plasticity, corpus callosum, musical training, pianists, string players, interhemispheric inhibition, fractional anisotropy, diffusion imaging

## Abstract

Long-term musical expertise has been shown to be associated with a number of functional and structural brain changes, making it an attractive model for investigating use-dependent plasticity in humans. Physiological interhemispheric inhibition (IHI) as examined by transcranial magnetic stimulation has been shown to be correlated with anatomical properties of the corpus callosum as indexed by fractional anisotropy (FA). However, whether or not IHI or the relationship between IHI and FA in the corpus callosum can be modified by different musical training regimes remains largely unknown. We investigated this question in musicians with different requirements for bimanual finger movements (piano and string players) and non-expert controls. IHI values were generally higher in musicians, but differed significantly from non-musicians only in string players. IHI was correlated with FA in the posterior midbody of the corpus callosum across all participants. Interestingly, subsequent analyses revealed that this relationship may indeed be modulated by different musical training regimes. Crucially, while string players had greater IHI than non-musicians and showed a positive structure-function relationship, the amount of IHI in pianists was comparable to that of non-musicians and there was no significant structure-function relationship. Our findings indicate instrument specific use-dependent plasticity in both functional (IHI) and structural (FA) connectivity of motor related brain regions in musicians.

## Introduction

Musical expertise is an important model for investigating training-related functional and structural alterations in the human brain. Musical training on most musical instruments involves the interaction between precise bimanual hand coordination and higher cognitive functions (Wan and Schlaug, 2010; Herholz and Zatorre, 2012) that typically requires fine-tuning over years of practice. Apart from functional brain changes as a consequence of musical training, several studies provide compelling evidence for structural and neurophysiological differences between musicians and non-musician controls (Jäncke, 2009).

For example, structural differences in the gray matter of auditory, frontal and motor cortices of musicians are thought to be related to enhanced information processing that has been linked to musical performance (Amunts et al., 1997; Gaser and Schlaug, 2003; Bermudez et al., 2009; Steele et al., 2013). As the primary white-matter tract connecting homologous cortical regions in the two hemispheres (Swinnen, 2002), the corpus callosum is thought to mediate the skilled bimanual coordination of expert musicians. Musicians tend to have larger anterior corpora callosa and more symmetric bimanual hand function than non-musicians (Schlaug et al., 1995). Musicians also have greater intrasulcal length of the precentral gyrus (Amunts et al., 1997) and enhanced fractional anisotropy (FA), a measure of white-matter organization, in the motor region of the corpus callosum (Steele et al., 2013). In parallel with these structural differences, there is evidence that musicians have decreased intracortical inhibition within (Rosenkranz et al., 2007) and interhemispheric (IHI) inhibition between (Ridding et al., 2000) motor cortices. However, the relation between these neurophysiological measures and brain structure in musicians remains largely unknown.

Few studies have attempted to link IHI with brain structure, and all have been performed in non-experts. Wahl et al. provided the first evidence linking greater IHI with enhanced FA in the motor region of the corpus callosum of young adults (Wahl et al., 2007). Fling and colleagues used an indirect measure of IHI, the ipsilateral silent period, to show similar results (Fling and Seidler, 2012; Fling et al., 2013), though the reverse may be true for older adults (Fling and Seidler, 2012).

There is cross-sectional evidence from expert musicians that different forms of long-term bimanual musical training may result in differences in both information processing (e.g., Margulis et al., 2009; Strait et al., 2012; Gebel et al., 2013) and brain structure (Bangert and Schlaug, 2006). Hence, the aim of the present study was to (i) investigate neurophysiological correlates of structural differences in the corpus callosum of musicians and non-musicians and (ii) determine potential differences in this relationship within subgroups of musicians that follow different bimanual training regimes (pianists and string players). We hypothesized that (a) IHI would differ between musicians and non-musicians and (b) individual differences in IHI would positively correlate with FA in the corpus callosum not only in non-experts but also in musicians. Furthermore, we hypothesized that this structure-function relationship would differ according to the amount of bimanual symmetric vs. asymmetric training. Therefore, we stratified musicians into pianists (relatively symmetric) and string players (more asymmetric) as these groups differ with respect to their individual training regimes and hand use.

## Materials and methods

Seventeen musicians (6 females) and sixteen non-musicians (10 females) were tested in this study. Musicians were currently performing musicians or musical students enrolled in a musical degree program. Non-musicians were selected such that they had no musical experience and were not performing repetitive hand skills such as professional typing and video gaming. All participants completed a musical experience questionnaire to determine the age of onset of training, length of formal training and hours of current practice (see Table 1 for participant demographic details). Participants were screened to ensure no contraindications for the use of transcranial magnetic stimulation (TMS) and magnetic resonance imaging (MRI). No participants were taking any medication at the time of the experiment. All participants were right-handed as assessed by the Edinburgh handedness inventory (Oldfield, 1971). The experimental protocol was approved by the local ethics committee of the University of Leipzig and participants provided written informed consent before participation. Initially, all participants received a structural MRI scan. On a separate day, interhemispheric inhibition (IHI) was assessed using paired-pulse TMS measurements. Analyses were performed on FA and IHI since both methods are established tools for assessing structural and functional plasticity in both musicians and non-musicians. More specifically, FA is a sensitive and reliable measure of white-matter structure that has previously been shown to be sensitive to experience-dependent plasticity in the corpus callosum (Steele et al., 2013). Since the corpus callosum connects both hemispheres, we used IHI, a well-established measure of functional connectivity, to directly assess interhemispheric communication between the primary motor cortices (Ferbert et al., 1992; Ridding et al., 2000).

**Table 1:** **Participant demographics**.

	**Non-musicians**	**Musicians**	**Pianists**	**String players**
*n*	16	15	7	8
Male/Female	6/10	9/6	4/3	5/3
Age (years)	23.81 (2.14)	24.13 (2.35)	24.29 (3.40)	24.00 (1.60)
**AGE OF ONSET (YEARS)**				
Mean (*SD*)	–	5.47 (1.25)	5.57 (1.62)	5.38 (0.92)
Range	–	4–8	4–8	4–6
**FORMAL TRAINING (YEARS)**				
Mean (*SD*)	–	11.40 (3.33)	10.00 (3.00)	12.63 (3.29)
Range	–	6–16	6–14	6–16
**CURRENT PRACTICE (H/WEEK)**				
Mean (*SD*)	–	6.60 (7.21)	4.00 (5.69)	8.88 (7.97)
Range	–	0–25	0–15	2–25
**ELECTROPHYSIOLOGY**				
1mV L (%)	45.38 (7.36)	46.67 (6.38)	49.00 (6.71)	44.63 (5.71)
1mV R (%)	47.50 (7.47)	47.07 (5.46)	49.14 (4.14)	45.25 (6.07)
IHI LR (%)	38.34 (24.57)^*^^+^	54.37 (15.49)	48.53 (16.34)	59.47 (13.67)
IHI RL (%)	45.22 (20.66)	43.82 (19.80)	42.56 (18.81)	44.93 (21.86)

[*] *denotes the significant difference in LR IHI between musicians and non-musicians (Kruskal-Wallis test, p < 0.05). There were no significant age differences (p = 0.70) between the groups nor any differences in age of onset (p = 1), formal training (p = 0.52), or current practice (p = 0.80) between pianists and string players*.

[+] *denotes the significant difference in IHI between string players and non-musicians (Bonferroni-corrected Kruskal-Wallis test, p < 0.05)*.

### Image acquisition

Whole-brain diffusion (60 directions, 7 *b* = 0 images, *TR* = 12900 ms, *TE* = 100 ms, *b* = 1000 mm/s^2^, 220 × 220 FOV, 1.7 mm isometric) and T1-weighted anatomical images (*TR* = 1.3 s, *TE* = 3.46 ms, 256 × 240 FOV, 1.0 mm isometric) were collected on a Siemens 3T Trio Tim equipped with a 32 channel head-coil. The anatomical scans of each individual were used for neuronavigated TMS using Brainsight (version 2, Rogue Research Inc., Montreal, Canada). Diffusion images were processed and analyzed with the tools available in the FMRIB Software Library (FSL 5.0.2) (Smith et al., 2004).

### Interhemispheric inhibition (IHI)

Dual coil paired-pulse TMS was used to assess the degree of interhemispheric inhibition (IHI) from the left (dominant) to right (non-dominant) primary motor cortex (M1) and vice versa. During the TMS session, participants were seated in an armchair with both arms relaxed and were instructed to keep their eyes open. Surface electromyography (EMG) was recorded bilaterally using surface Ag/AgCl electrodes positioned on the skin overlying the first dorsal interosseous (FDI) of both hand muscles in a bipolar montage. The signal was amplified using an EMG device (D360 8-channel amplifier, Digitimer Ltd., Welwyn Garden City, Hertfordshire, UK) with band pass filtering between 50 and 2000 Hz. The signal was digitized at a frequency of 5000 Hz (CED Power 1401, Cambridge Electronic Design, Cambridge, UK) and fed off-line to a data acquisition system (Signal Version 4.02, Cambridge Electronic Design, Cambridge, UK) for further analysis. The absence of voluntary contraction during the IHI measurements was monitored online by visual inspection of the EMG signal and off-line by inspection of each individual trace. Trials with background EMG were excluded from the analysis.

### Neuronavigated TMS

Each participant's MRI scan was used to individually monitor the position of both TMS coils. The target regions were the left and right M1, which were identified by applying supra-threshold single-pulse TMS at rest using a 70-mm figure-of-eight-coil connected to one of two Magstim 200^2^ stimulators (Magstim Co., Whitland, UK). As reported previously (Wahl et al., 2007), for IHI measurements from left to right M1, the conditioning coil was placed tangentially over the left M1, with the handle pointing laterally to induce a lateral-to-medial current. The test coil was placed over right M1, with the handle pointing backward and 45° away from the midline to induce a current from lateral/posterior to medial/anterior. For IHI from right to left, the coil position was reversed. The area underneath the coil where a single TMS pulse elicited the most consistent motor evoked potentials (MEP) with the highest amplitude of the contralateral FDI was marked as the hot spot for M1. Before the measurement of IHI the test stimulus (TS) and conditioning stimulus (CS) were set to an intensity that evoked MEPs of approximately 1 mV peak-to-peak amplitude. For IHI, the mean MEP amplitudes obtained in response to 20 consecutive randomized TS and CS+TS conditions were recorded and processed for further analysis. In accordance with previous work on musicians, we chose an interstimulus interval of 10 ms in the CS+TS condition (Ridding et al., 2000). IHI was defined as the percentage difference in MEP size in the CS+TS relative to the TS condition using the following formula: IHI [%] = [1 − meanCS + TS / meanTS] × 100.

### Assessing group differences in IHI

An initial One-Way analysis of variance (ANOVA) was used to test for the main effect of group (musicians × non-musicians). If the data was not normally distributed, non-parametric Kruskal-Wallis tests were conducted. Subsequent Bonferroni-corrected pairwise comparisons were used to clarify effects where necessary. All tests were conducted within SPSS (v22, IBM), and considered statistically significant when the *p*-value was below 0.05.

### Diffusion imaging

Diffusion images were first motion corrected and corrected for eddy-current distortions prior to creating voxelwise maps of diffusion parameters. Individual FA maps were then nonlinearly registered to the FMRIB58_FA standard space template, minimally smoothed (σ = 2 mm), and then averaged to create the group mean FA map. This map was used to create a mask of the corpus callosum in the following manner. A binary mask was first manually drawn over the entire corpus callosum from *x* = −4 to *x* = 4, and a whole-brain binarised FA > 0.20 mask was created from the mean FA image to exclude most gray-matter regions. The two masks were combined to create the final corpus callosal mask that was used in the correlational analyses. All imaging analyses included both age and sex as covariates of no interest, and were fully corrected for multiple comparisons at α = 0.05. Correlations were assessed for significance using nonparametric permutation testing with 5000 permutations and threshold-free cluster enhancement (TFCE) with FSL randomize (Smith and Nichols, 2009). Regions identified in the FA correlations were used as masks to extract radial, axial, and mean diffusivity for description and additional analyses as necessary.

### Assessing the relation between IHI and the corpus callosum

As a first step, we performed a correlation analysis between the amount of individual IHI and *FA* values throughout the volume of the corpus callosum for all participants (musicians and non-musicians) using FSL randomize and TFCE with 5000 permutations. We subsequently extracted mean *FA* values for any significant finding within the corpus callosum. Based on these *FA* values, we performed separate Pearson's correlation analyses on each group (musicians and non-musicians) within SPSS. Axial and radial diffusivity measures extracted from the same regions were used to further clarify findings as related to diffusivity along (axial) or perpendicular to (radial) the predominant fiber direction.

Based on previous research, we expected to find a significant positive correlation between IHI and FA in the posterior midbody of the corpus callosum in non-experts (Wahl et al., 2007). Therefore, we first attempted to replicate these findings with a planned one-tailed Pearson's correlation between mean FA extracted from the significant region and IHI within non-musicians. Subsequently, we asked whether a similar relationship could also be observed in musicians. To be as conservative as possible, additional correlation analyses for musicians and musician subgroups (pianists and string players) were performed using two-tailed correlations with age and sex as covariates of no interest.

We also performed additional analyses within the musicians group to determine if measures of musical training would be related to FA in similar regions of the corpus callosum. To accomplish this, we correlated FA throughout the volume of the corpus callosum with the age of onset, years of formal training and hours of current practice in musicians in separate analyses with FSL randomize (5000 permutations with TFCE, α = 0.05). The first analysis tests for the overall relationship between IHI and FA in the corpus callosum while the second investigates the potentially mediating effects of musical training on the relationship.

## Results

### Demographics

Most musicians in our study began training before the age of 7 (range of 4–8), though there was a large range of formal training (6–16 years) and current hours of practice (0–25 h/week). There were no significant differences in age between musicians and non-musicians (*p* = 0.70), nor were there any demographic differences between pianists and string players (age: *p* = 1, age of onset: *p* = 1, formal training: *p* = 0.52, current hours of practice: *p* = 0.80). A detailed breakdown of participant demographics and TMS measures is provided in Table 1.

### Interhemispheric inhibition in musicians and non-musicians

**Figure 1:**
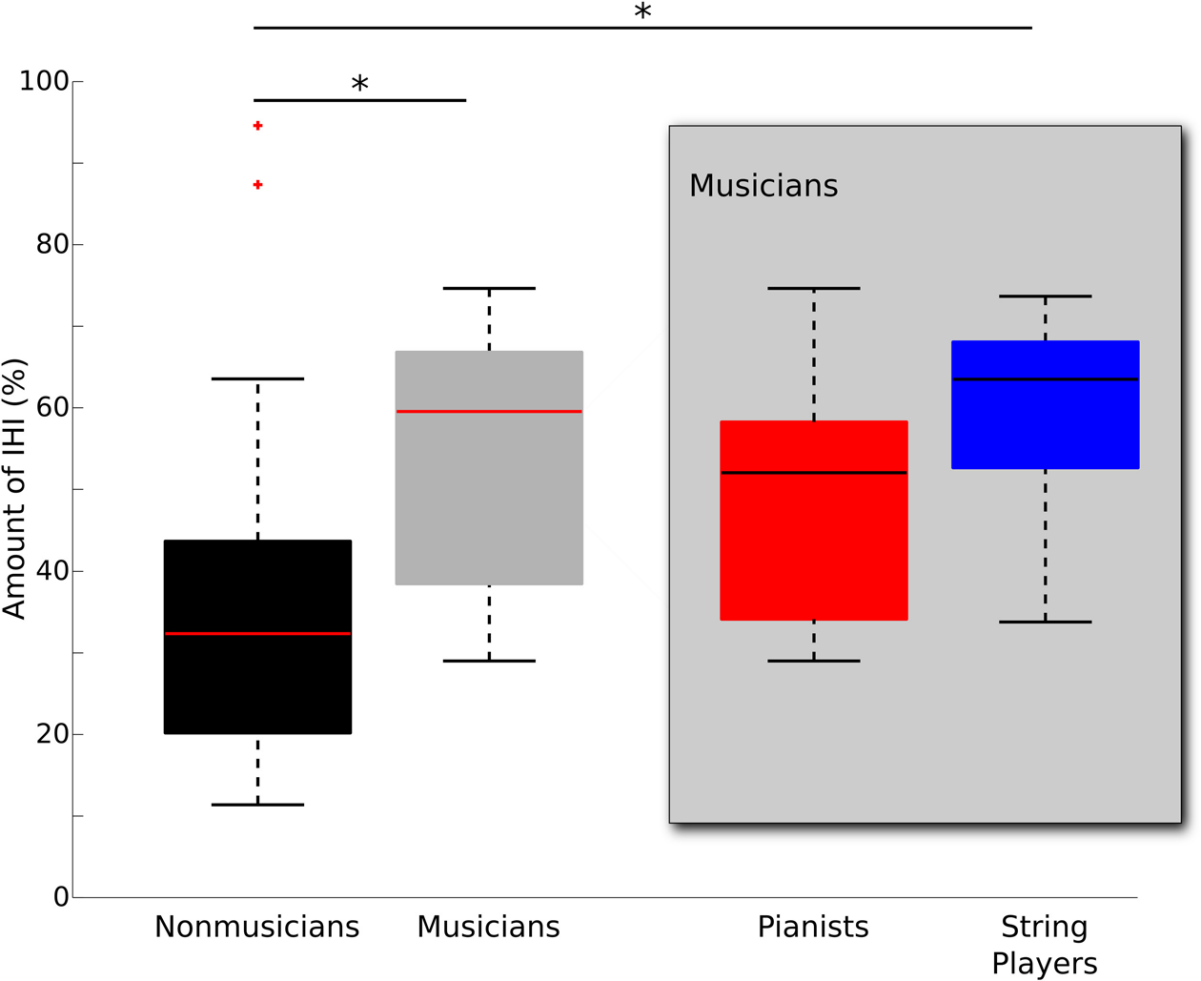
**Median boxplot of group differences in IHI from left to right M1 between musicians (gray) and non-musicians (black)**. On each box, the horizontal line represents the median, the upper and lower edges the 75th and 25th percentile, the whiskers show the range, and red “+”s indicate statistical outliers. Musicians were subdivided into pianists (*n* = 7) and string players (*n* = 8) and plotted separately within the gray box. Significant group differences are denoted with ^*^(*p* < 0.05, corrected for multiple comparisons).

The mean threshold intensity required to evoke a 1mV MEP response did not differ between musicians and non-musicians in either the left or right M1 (left M1: *p* = 0.61; right M1: *p* = 0.85). There was a significant difference in left to right IHI (Kruskal-Wallis non-parametric test, *p* < 0.05). Musicians had stronger IHI from left to right M1 as compared to non-musicians (mean | median and standard deviation for musicians (M) = 54.37 | 59.53 ± 15.49%, non-musicians (NM) = 38.34 | 32.36 ± 24.57%; Table 1). To explore whether or not this effect differed by the different task demands of particular instruments, we subdivided musicians into pianists (*n* = 7) and string players (*n* = 8) for subsequent pair-wise comparisons. Paired comparisons revealed greater IHI in string players than non-musicians (*p* < 0.05). Pianists did not differ from either non-musicians (*p* = 0.72) or string players (*p* = 0.80) (mean | median and standard deviation of IHI for pianists = 48.53 | 52.03 ± 16.34%; string players = 59.47 | 63.48 ± 13.67%; non-musicians = 38.34 | 32.36 ± 24.57%). Interestingly, no such differences in IHI could be observed when investigating transcallosal inhibition from right to left M1 (*p* = 0.92; mean and standard deviation for musicians 43.82 ± 19.80%; non-musicians 45.22 ± 20.66%). Figure 1 depicts the spread and group medians of the groups. Table 1 lists the musician demographics for each subgroup.

### Diffusion weighted imaging (DWI): the relationship between IHI and FA

**Figure 2:**
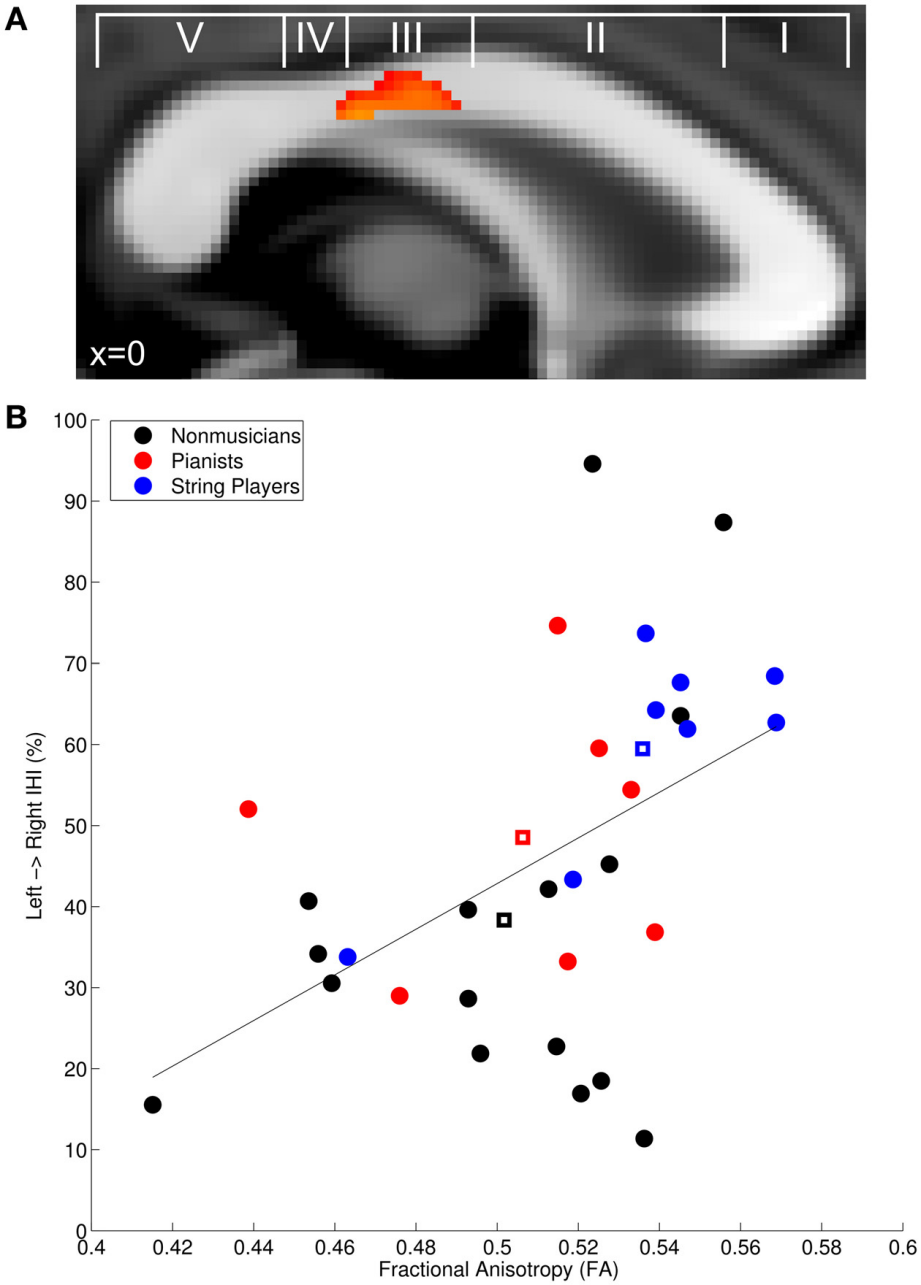
**(A)** Fractional anisotropy in the posterior midbody of the corpus callosum was positively correlated with baseline IHI across all participants. **(A)** is overlayed with the classification scheme of Hofer and Frahm (2006). **(B)** Mean FA extracted from the significant region plotted against IHI. Non-musician controls are depicted in black, pianists in red, string players in blue, and group centroids are denoted with squares. The solid line depicts the linear regression through the plotted points. Group-wise correlations performed on the extracted *FA* values revealed that the positive correlation is also present within non-musicians (*r* = 0.44, *p* < 0.05), musicians (*r* = 0.51, *p* < 0.05), and string players (*r* = 0.96, *p* < 0.005).

We correlated left to right IHI with FA in the corpus callosum across all participants to determine how FA is linked to transcallosal information processing. In accordance with our hypothesis, there was a significant positive correlation in the posterior midbody of the corpus callosum (*t*-max = 3.92; Figure 2A), which is known to connect the left and right primary motor cortices (Hofer and Frahm, 2006; Chao et al., 2009; Fling et al., 2013; Steele et al., 2013). Mean diffusion measures from this region were extracted to plot the observed relationship across musicians and non-musicians (Figure 2B) and to further characterize the finding. The positive correlation with FA was primarily driven by a negative correlation with radial diffusivity (RD: *r* = −0.49, *p* < 0.05) rather than axial diffusivity (AD: *p* = 0.18). There was also a significant negative correlation with mean diffusivity in this region (MD: *r* = −0.47, *p* < 0.05).

Replicating previous work, the planned correlation between left to right IHI in non-musicians and extracted FA revealed the expected significant positive correlation (NM: *r* = 0.44, *p* < 0.05) (Wahl et al., 2007). Musicians also showed a significant positive correlation between IHI and FA (M: *r* = 0.51, *p* < 0.05), suggesting that the same relationship exists for musical expertise. However, to determine whether the effect within musicians was driven by instrument type, we again subdivided musicians into pianists and string players. Interestingly, string players showed a significant positive correlation (*r* = 0.96, *p* < 0.005) while pianists did not (*r* = −0.52, *p* = 0.37). The difference between these correlations was statistically significant (Fisher's *r*-to-*z* transform: *z* = 3.76, *p* < 0.001), providing additional evidence that different training regimes may result in different structure-function relationships.

### Age of onset, formal training, and practice

**Figure 3:**
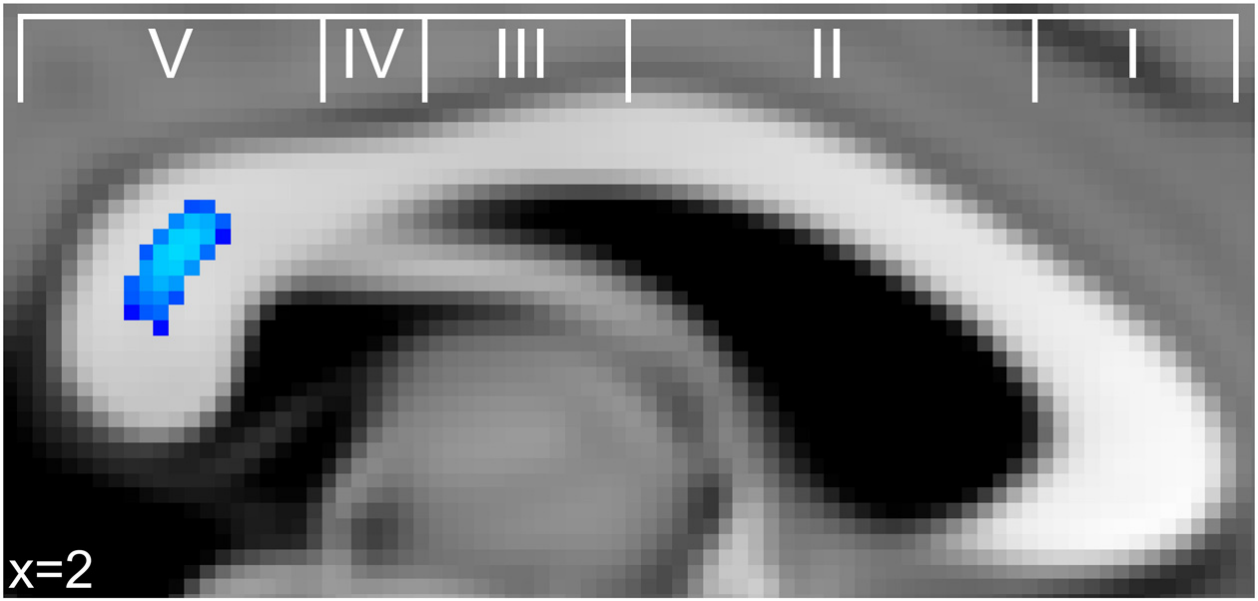
**Significant correlations between FA in the corpus callosum and current practice (blue) across musicians in the splenium of the corpus callosum**. This finding is in a similar region of the splenium where FA significantly correlated with retrospective hours of musical practice during adolescence in a study by Bengtsson et al. (2005).

An additional set of correlations between musicianship variables and FA was conducted to test the hypothesis that FA in the same region identified in the IHI analysis was also related to measures of musical training. We found no indication of a correlation between any training variable and FA in the posterior midbody of the corpus callosum. There was, however, a significant positive correlation between current hours of practice and FA in the splenium (Figure 3) across regions linking temporal, somatosensory, and occipital cortices (Hofer and Frahm, 2006; Chao et al., 2009).

## Discussion

The current study assessed white-matter structure of the corpus callosum as a marker for interhemispheric information processing in musicians and non-musicians. It replicates and builds upon recent work in non-experts suggesting that microstructure of the corpus callosum can be linked with functional connectivity measures within the motor system (Wahl et al., 2007). As a marker for transcallosal information processing, we compared differences in the amount of interhemispheric inhibition between musicians and non-musicians. Overall, we found that musicians show stronger left to right IHI than non-musicians. Furthermore, we provide novel evidence that experts in bimanual control (musicians) show a structure-function relationship similar to that observed in non-experts. Crucially, more detailed analyses comparing subgroups of musicians (pianists and string players), revealed that this relationship may at least partially depend upon different training regimes. Here the structure-function relationship was mainly driven by string players, the sub-group who exhibited stronger IHI than non-musicians. In contrast, the amount of IHI in pianists was comparable to that of non-musicians and was not correlated with white-matter structure. Thus, we provide novel evidence that even though the structure-function relationship between IHI and FA exists across musicians and non-musicians *in general*, it may not be *generalizable* to every individual musician.

### Intracortical and interhemispheric alterations in musicians

There is evidence that musicians show higher motorcortical excitability (Rosenkranz et al., 2007), reduced surround intracortical inhibition (Shin et al., 2012), and reduced intracortical inhibition and facilitation (Nordstrom and Butler, 2002). However, knowledge about alterations in interhemispheric inhibition (IHI) between both primary motor cortices of musicians remains sparse. Until now, only a single study investigated this in a relatively small cohort of musicians. Ridding et al. found that professional musicians had significantly *lower* IHI than non-musicians (Ridding et al., 2000).

In contrast, musicians in our study had, on average, significantly *stronger* IHI when compared to non-musicians, an effect that was predominantly driven by string players. These apparently contradictory results could potentially be due to the differences in expertise and practice schedules of the respective musicians, especially since it has already been shown that short periods of bimanual practice affects the amount of IHI (Shim et al., 2005). Musicians in our study had a wide range of hours of practice while Ridding and colleagues tested professionals with a high and narrow range of practice (17.5–28.0 h per week), indicating substantial differences in the amount of training. There is also no appreciable difference in the reported IHI of non-musician controls between the two studies (~35% reduction in both)—suggesting that differences between the two studies are unlikely to be caused by differences in TMS methodology. However, interpreting differences between our results and this proof-of-concept work should still be done with caution, especially since Ridding and colleagues pooled their IHI measurements (right to left and left to right IHI).

Beyond this simple group comparison (musicians vs. non-musicians), we provide novel evidence that the amount of IHI may be differentially modulated by individual practice regimes in musicians. While we did not find evidence that pianists show altered IHI as compared to non-musicians, IHI in string players was significantly larger. Thus, we hypothesize that differences in IHI across musicians might depend upon instrument and/or individual practice regimes. For example, pianists move their upper limbs both independently and in a more synchronized fashion when playing (Haslinger et al., 2004). Here, greater synchronization may be reflected by more refined cortical motor representations that require less functional activity than in untrained controls (Jäncke et al., 2000; Krings et al., 2000; Meister et al., 2005). String players, on the other hand, move their arms, hands, and fingers in almost completely different ways when performing (Baader et al., 2005). The non-dominant hand is concerned with fingering the strings while the other controls the movements of the bow. Therefore, IHI in musicians may be modulated by the particular bimanual demands of the instrument on which they train.

### The relationship between IHI, the corpus callosum, and bimanual expertise

Our findings are consistent with recent work demonstrating a structure-function relationship in the corpora callosa of non-experts with non-invasive measures of functional connectivity such as paired-pulse TMS at rest (IHI, Wahl et al., 2007) and single-pulse TMS during contraction of the ipsilateral hand (iSP, Fling and Seidler, 2012; Fling et al., 2013). This relationship was predominantly observed in the posterior midbody of the corpus callosum, which consists of transcallosal fibers directly connecting the primary motor cortices (Hofer and Frahm, 2006; Wahl et al., 2007). Here, we confirm the findings of these studies in non-experts and extend them by showing that a similar relationship can also be observed in musicians.

Our results also complement recent research linking FA in the corpus callosum to bimanual motor performance. Reduced FA has been linked to decreased bimanual performance in sufferers of multiple sclerosis (Bonzano et al., 2008), adolescents (Muetzel et al., 2008), and young adults (Johansen-Berg et al., 2007; Sisti et al., 2012). Consistent with our findings, those studies that assessed bimanual tapping performance in young adults also identified correlations with FA in the posterior midbody of the corpus callosum (Johansen-Berg et al., 2007; Fling and Seidler, 2012). However, these findings are not without controversy. Using different bimanual tasks, Fling and colleagues found a *negative* correlation between FA and bimanual performance in young adults (Fling et al., 2011; Fling and Seidler, 2012) but a *positive* correlation in older adults (Fling and Seidler, 2012) while Johansen-Berg et al. identified a *positive* correlation in young adults (2007). Therefore, both the type of bimanual task and participant demographics, such as age, may be important factors affecting the link between performance and FA.

Previous work has also established a link between corpus callosum structure and musicianship. The anterior portion of the corpus callosum has been shown to be larger in musicians, particularly in those who began training before the age of seven (Schlaug et al., 1995). Earlier onset of musical training has also been linked to enhanced FA in this region (Imfeld et al., 2009; Steele et al., 2013). In addition, FA in the splenium of musicians has been linked to hours of practice in both the current study and previous work (Bengtsson et al., 2005). There is also evidence that the onset of musical training during childhood (Steele et al., 2013) and musical practice (Hyde et al., 2009) can differentially affect corpus callosum structure in the posterior midbody. Our study confirms this research in musicians and is consistent with evidence linking bimanual performance to FA in the same region (Johansen-Berg et al., 2007; Fling and Seidler, 2012). Furthermore, it suggests that IHI and its relationship to FA could be differentially affected by different bimanual training regimes.

Though few studies have directly compared different groups of musicians, there is some evidence for functional (Gebel et al., 2013), structural (Bangert and Schlaug, 2006), and neurophysiological differences between musicians that are tuned to specific instruments (Margulis et al., 2009; Strait et al., 2012). Crucially, Bangert and Schlaug provide evidence that the motor knob in the left hemisphere tends to be more developed in professional pianists (where the left hand can often play a supporting role) while the motor knob in the right hemisphere tends to be more developed in professional string players (where the more demanding fingering movements are made with the left hand). Furthermore, it has been hypothesized that bilateral motor cortex size may be more symmetrical in pianists (Amunts et al., 1997) and that string players have enhanced cortical representation for the left hand (Elbert et al., 1995). Together with the current study, these findings indicate that the type of instrument played may have a long-term effect on neurophysiology and, ultimately, its link to white-matter structure in the corpus callosum.

### Future challenges

Even though we cannot completely rule out the potential effects of pre-existing differences between and within our study sample, there are several lines of evidence that suggest FA in this region undergoes training-related plasticity. FA in this region may be under less genetic control than other regions (Chiang et al., 2009), can be influenced by the age of onset of musical training (Steele et al., 2013), and several months of musical training can lead to structural changes in an overlapping region of the corpus callosum (Hyde et al., 2009). In light of the present results, future studies utilizing musicians as an expert population should attempt to characterize and control for the differences present in subgroups of musicians.

### Relevance

In many scientific studies, musicians are regarded as a model for use-dependent plasticity without taking into account the differences in training regimes that may exist across musicians and musical instruments. The novelty of the present study was that we observed musical training-related functional and structural differences, and that this relationship was influenced by the type of instrument and/or training regimes. The fact that such structure-function relationships both exist and can differ depending on the specific type of training has important implications for neurorehabilitation. This is particularly relevant for rehabilitation protocols that utilize musical training as an adjuvant strategy to promote recovery from functional deficits (e.g., Rodriguez-Fornells et al., 2012). Given our findings that asynchronous bilateral training in string players promotes the strongest structural and functional brain alterations, it seems reasonable to hypothesize that asynchronous bilateral training may be a particularly powerful tool to promote brain plasticity and behavioral recovery. However, in this context it is still an open question whether asynchronous or synchronous rehabilitative training regimes are more or less effective for the treatment of *specific* functional deficits.

## Conclusion

To our knowledge, this is the first study to combine a neurophysiological measure of interhemispheric inhibition with an investigation of white-matter structure in experts (musicians) and non-experts in bimanual control. We found that musicians had stronger IHI than non-musicians and a significant positive correlation between IHI and FA in the posterior midbody of the corpus callosum. This extends previous research in non-experts and provides novel evidence for a similar relationship in experts in bimanual control. Crucially, we provide evidence that the type of instrument played, and thus different musical training regimes, may also influence IHI and FA in the corpus callosum—suggesting that there is a specific link between the mode of bimanual training, neurophysiology, and brain structure.

### Conflict of interest statement

The authors declare that the research was conducted in the absence of any commercial or financial relationships that could be construed as a potential conflict of interest.
